# A case of cutaneous *Scedosporium apiospermum* infection in a patient on a janus kinase inhibitor

**DOI:** 10.1002/ski2.188

**Published:** 2023-03-10

**Authors:** Louis J. Born, Shealinna Ge, Juris P. Germanas

**Affiliations:** ^1^ University of Maryland School of Medicine Baltimore Maryland USA; ^2^ Department of Dermatology University of Maryland Medical Center Baltimore Maryland USA; ^3^ Department of Dermatology Veterans Affairs Medical Center Baltimore Maryland USA

## Abstract

Scedosporium apiospermum is a mold that is usually found in soil and polluted water, but has also been linked to contaminated ambient air in hospitals. This fungus typically behaves as a rare opportunistic pathogen affecting immunocompromised patients in whom disseminated disease can readily occur, causing shock and multiorgan failure. We report the first case of cutaneous Scedosporium apiospermum infection in a patient with rheumatoid arthritis treated with a Janus kinase inhibitor. We also reviewed other cutaneous manifestations of Scedosporium apiospermum reported between 2003 and 2022.

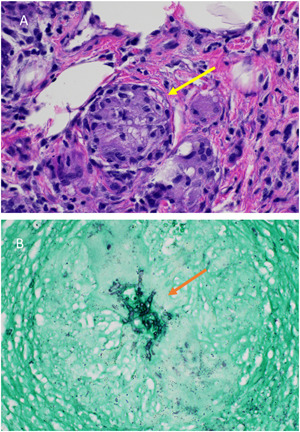

1

## ETHICS STATEMENT

Not applicable.

Dear Editor,

1

*Scedosporium apiospermum* is a mold that causes many types of infections, particularly in immunocompromised hosts. This fungal organism is typically found in soil or polluted water, and infections in humans result from inhalation or direct inoculation from trauma.^1^ There have been reported cases involving the respiratory tract, central nervous system, eye, skin, soft tissue, and bone.^2^ Immunocompetent patients typically have superficial or localized infection of the skin or cornea,^3^ whereas immunocompromised patients can manifest hematogenous dissemination affecting multiple organ systems.^4^

On the skin, lesions typically appear as nodules or erythematous to violaceous papules or bullae with woody induration.^5^ Mycetomas have been reported in immunocompetent patients after trauma.^5^ The majority of cutaneous manifestations, however, affect immunocompromised patients in whom no trauma was reported. We report here the first case of this organism causing an opportunistic cutaneous infection in a patient on a Janus kinase (JAK) inhibitor, and review other cutaneous manifestations of *Scedosporium apiospermum* reported between 2003 and 2022.

**FIGURE 1:**
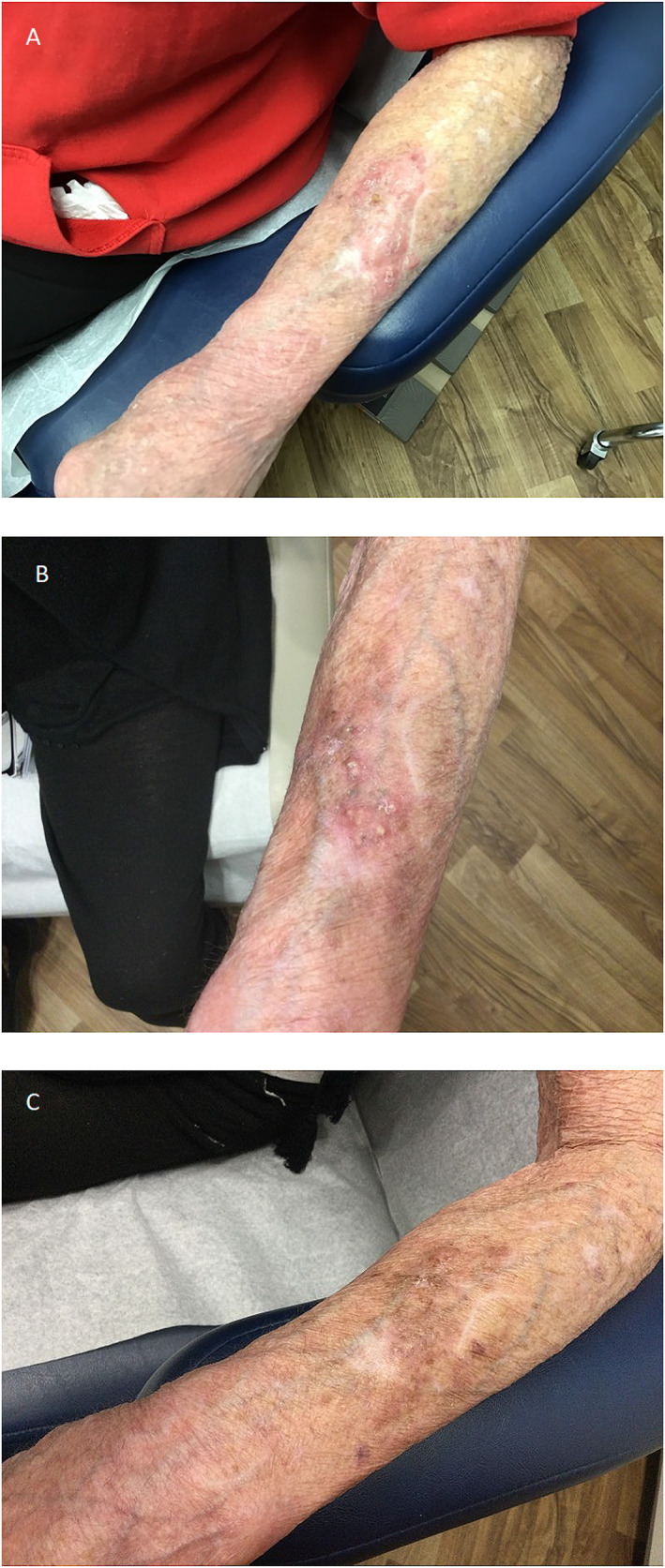
Clinical photographs of affected arm of patient (a) at initial presentation (b) 1 month after itraconazole treatment and discontinuation of tofacitinib (c) 2 months after itraconazole treatment and discontinuation of tofacitinib.

**FIGURE 2:**
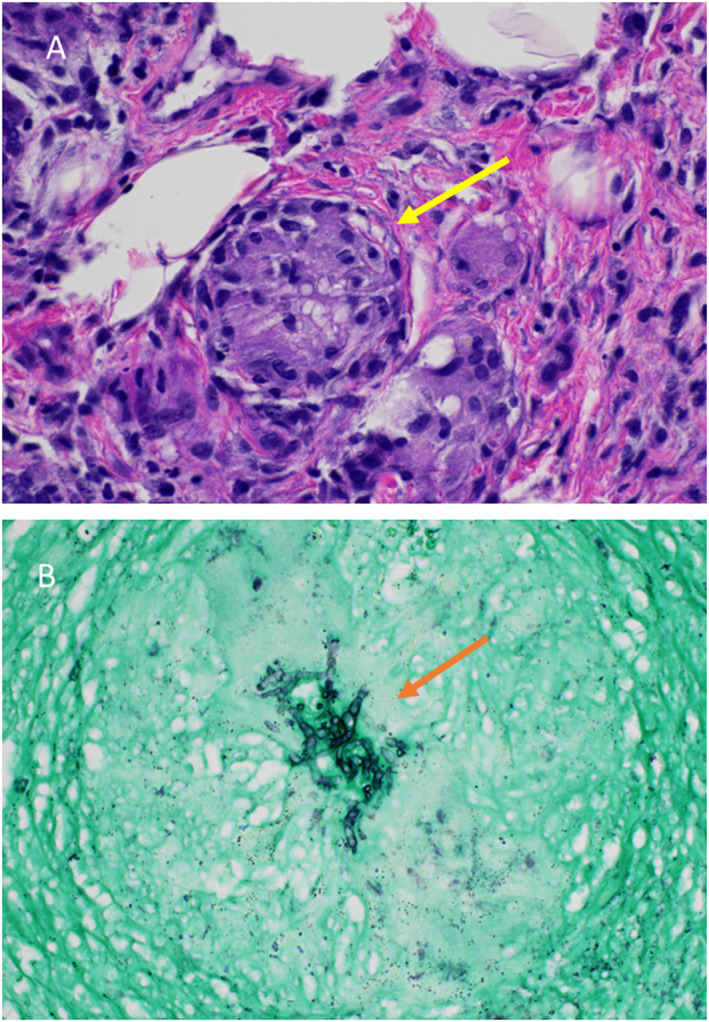
Histologic specimen taken before treatment (a) 200 × Magnification hematoxylin‐eosin‐stained specimen demonstrating granulomas (yellow arrow) and lymphohistiocytic infiltrate (b) 400 × Magnification GMS‐stained specimen revealing focal collection of septate hyphae (orange arrow) within a giant cell.

A 72‐year‐old woman with a history of rheumatoid arthritis on tofacitinib 10 mg once daily, presented with a 1‐month history of an enlarging, itchy, nodular plaque on the left forearm (Figure 1a). A skin biopsy revealed extensive chronic and granulomatous inflammation, with multinucleated giant cells and areas of necrosis (Figure 2a). While an AFB stain was negative for mycobacteria, a GMS stain highlighted a focal collection of septate hyphae within a giant cell (Figure 2b). A fungal culture identified the causative organism as *Scedosporium apiospermum*.

On follow up examination, scaly violaceous papules and furuncles on the left extensor forearm were evident. Tofacitinib was discontinued and antifungal therapy was initiated. While the antifungal agent with the most evidence of effectiveness against Scedosporium infections is voriconazole, itraconazole 100 mg once daily was started due to formulary constraints.^6^ Upon reevaluation 1 month later, interim improvement in the number of nodules was noted, but induration at the proximal and distal edges persisted (Figure 1b). The patient continued the same regimen for an additional month, at which time almost complete flattening of the nodules was observed (Figure 1c).

A literature review for reported cases between 2003 and 2022 found a total of 33 instances, including our own, reporting cutaneous *Scedosporium apiospermum* infections. Table 1 lists these cases and their clinical features and outcomes. While many factors determine the publication rate of articles concerning specific diseases, the low number of cases (1.7 cases annually), suggests that cutaneous *Scedosporium apiospermum* infections are fairly rare. Only three patients were immunocompetent, and all three cases resulted after trauma to the skin. The remaining cases were in immunocompromised patients, including transplant recipients, previous diagnoses of leukemia, and those on immunosuppressive agents for diseases, such as rheumatoid arthritis, ulcerative colitis, and temporal arteritis.

**TABLE 1:** Reported cases of *Scedosporium apiospermum* between 2003 and 2022 with associated clinical features, treatment, and outcomes

Ref	Age/Sex	Underlying medical condition	Immunosuppressive agent(s)	*S. Apiospermum* treatment	Outcome
Ishii S, et al. 2015^7^	77/F	Idiopathic interstitial pneumonia	Cyclosporine	Itraconazole after removal of nodes	No recurrence after 2 years follow up
Goldman C, et al. 2016^8^	77/M	Temporal arteritis	Prednisone	IV voriconazole, then PO voriconazole, prednisone tapered off completely by day 84. Readmitted and IV micafungin, IV GM‐CSF started.	Multiple readmission. Death on day 266
Toth E, et al. 2017^9^	70/M	Nephrotic syndrome	Corticosteroids	IV Fluconazole, PO terbinafine, and antimicrobial agents (i.e., betadine, hydrogen peroxide, and boric acid powder). Steroid therapy dose gradually decreased.	Asymptomatic and currently maintains a low dose of corticosteroid therapy.
Harrison M, et al. 2012^10^	59/W	Myelodysplastic syndrome (in remission from chronic lymphocytic leukemia)	N/A	IV voriconazole, then placed in comfort care 3 days after admission	Death 3 days after admission
Sakata Y, et al. 2018^11^	77/M	Rheumatoid arthritis	Betamethasone and tacrolimus	PO voriconazole and local hyperthermia. Then PO itraconazole and terbinafine started due to hepatotoxicity.	Recovered completely after 12 months of treatment
Boyce Z, et al. 2013^12^	85/M	Meningioma with accompanying cerebral edema	Dexamethasone	Topical dilute potassium permanganate soaks and PO itraconazole. Then PO voriconazole after sensitivities resulted and lack of clinical improvement with itraconazole.	Full resolution of symptoms
Stoneham A, et al. 2017^13^	47/M	Renal transplant (first, failed; second, failing)	Prednisolone, tacrolimus, and mycophenolate mofetil	Surgical resection and PO voriconazole.	Wound healed and no evidence of recurrence
Makino K, et al. 2011^14^	28/F	Ulcerative colitis and aortitis	Prednisolone and cyclosporin	Resection and PO itraconazole.	Cured of infection
Stur‐Hofmann K, et al. 2009^15^	61/M	Chronic obstructive pulmonary disease	Prednisolone	PO voriconazole. Systemic steroid therapy for COPD switched to monotherapy with inhalative beta‐2‐sympathomimetics.	90% reduction and improvement by 6 weeks, and clinically undetectable by 3 months. Treatment was completed after 6 months.
Boyd M, et al. 2018^16^	63/M	Heart transplant	Mycophenolic acid, prednisone, and tacrolimus	PO voriconazole (delays in treatment were encountered due to multiple issues including patient being lost to follow‐up and drug cost)	Skin lesions resolved on day 516.
Shinohara M, et al. 2009^17^	60/M	Bilateral lung transplant	N/A	PO voriconazole	Skin lesions resolved over 8 months
Azofra M, et al. 2010^18^	65/F	SLE/SLE‐induced immune thrombocytopenia	Oral prednisone and azathioprine	Patient opted for surgical debridement and intralesional injection of voriconazole versus sPO voriconazole due to hepatotoxicity side effect.	Lesion completely healed after 3 months of treatment.
Mays R, et al. 2012^19^	80/F	Rheumatoid arthritis	Abatacept (orencia)	PO terbinafine and debridement of remaining lesions	Resolved after surgical debridement
Kollu V, et al. 2021^20^ ^a^	58/M	N/A	N/A	PO voriconazole	Appropriate clinical improvement after 6 months
Kim J, et al. 2014^3^ ^b^	75/M	N/A	N/A	PO itraconazole and terbinafine	Lesions subsided but left a scar
Takeuchi M, et al. 2011^21^	62/F	Recurrent idiopathic thrombocytopenic purpura and diffuse large b‐cell lymphoma	R‐CHOP	PO itraconazole, but no improvement. Then voriconazole with pus drainage by finger compression twice every day.	Resolved with normal epidermis after 2 months of voriconazole treatment.
Strunk T, et al. 2015^22^	57/M	Kidney transplant	Tacrolimus and prednisolone	PO voriconazole and surgical excision of nodules.	Wound healed and no evidence of recurrence
Yoneda K, et al. 2011^23^	75/F	Stage III lung squamous cell carcinoma	Docetaxel, carboplatin, and oral prednisolone	PO voriconazole	Skin lesions healed over the 2 months of therapy, but died of hemoptysis
Gupta M, et al. 2013^24^	45/M	N/A	N/A	PO Fluconazole	Failed to follow up
Perez F, et al. 2019^25^ ^c^	55/F	Kidney transplant	Tacrolimus, mycophenolate mofetil, and methylprednisolone	IV voriconazole then oral voriconazole as outpatient	Resolved with no evidence of new signs of infection,
Ezzedine K, et al. 2008^26^	65/F	Kidney transplant	Cyclosporin, mycophenolate mofetil, and corticosteroids	Final therapy consisted of PO voriconazole with reduction of cyclosporin and complete withdrawal of mycophenolate mofetil.	Two recurrences occurred due to halting of voriconazole therapy after hepatic cytolysis. Patient currently in remission for 8 months on final adjusted therapy.
Yu Z, et al. 2012^27^	23/M	Acute myelogenous leukemia	Daunorubicin + Cytarabine AND Mitoxantrone + Cytarabine	PO voriconazole and topical terbinafine hydrochloride.	Lesion resolved and became covered with normal epidermis.
Company‐Quiroga J, et al. 2018^28^	82/M	Chronic lymphocytic leukemia	N/A	PO voriconazole. Systemic steroid therapy for COPD switched to monotherapy with inhalative beta‐2‐sympathomimetics.	Lesions disappeared after 22 days of treatment
Braud A, et al. 2019^29^	86/M	Cerebral disease secondary to melanoma	Prednisone	PO voriconazole	Favorable progression
Duretz C, et al. 2017^30^	67/M	Bilateral lung transplant	Tacrolimus and methylprednisolone	IV voriconazole and reduction of tacrolimus dosage.	Died 7 days after admission
Tsuji G, et al. 2016^31^	62/M	Rheumatoid arthritis	Prednisone and methotrexate	N/A	Died 1 week after dermatological consult
Tsuji G, et al. 2016^31^	60/M	Chronic kidney failure	N/A	PO voriconazole	Died 1 week after starting treatment due to multiple organ failure
This study	72/F	Rheumatoid arthritis	Tofacitinib (Xeljanz XR)	PO itraconazole	Appropriate clinical improvement after 2 months

^a^ Co‐infected with Neisseria spp.

^b^ Co‐infected with *Mycobacterium chelonae*.

^c^ Co‐infected with Corynebacterium.

In our review, we found that successful treatment has been reported with voriconazole, fluconazole, and itraconazole. In a majority of these anti‐fungal regimens, reduction or absolute withdrawal of the immunosuppressive agent was also completed until resolution of the infection. Surgical management was also used in six cases.

In summary, *Scedosporium apiospermum* is rare opportunistic fungal pathogen most commonly affecting immunocompromised patients, in whom disseminated disease leads to increased mortality. Early detection of this organism, discontinuation of immunosuppressive agent, and treatment with a systemic antifungal agent, is critical for positive outcome.

## CONFLICT OF INTEREST

None to declare.

## AUTHOR CONTRIBUTIONS

**Louis J. Born**: Data curation (Lead); Writing – original draft (Lead). **Shealinna Ge**: Data curation (Equal); Writing – original draft (Supporting). **Juris P. Germanas**: Conceptualization (Lead); Investigation (Equal); Supervision (Lead); Writing – review & editing (Lead).

## FUNDING INFORMATION

This article received no specific grant from any funding agency in the public, commercial, or not‐for‐profit sectors.

## Data Availability

Data sharing not applicable—no new data generated, or the article describes entirely theoretical research.
